# Chronic liver diseases and erectile dysfunction

**DOI:** 10.3389/fpubh.2022.1092353

**Published:** 2023-01-06

**Authors:** Guanghui Zang, Xv Sun, Yufeng Sun, Yan Zhao, Yang Dong, Kun Pang, Ping Cheng, Meng Wang, Yuli Zheng

**Affiliations:** ^1^Department of Urology, Xuzhou Central Hospital, Xuzhou, Jiangsu, China; ^2^Graduate School, Bengbu Medical College, Bengbu, Anhui, China; ^3^Department of Cardiology, Xuzhou Central Hospital, Xuzhou, Jiangsu, China

**Keywords:** chronic liver disease, erectile dysfunction, risk factors, testosterone, chronic hepatitis

## Abstract

Chronic liver diseases (CLDs) are characterized by progressive necrosis of hepatocytes, which leads to liver fibrosis and cirrhosis, and ultimately liver dysfunction. The statistics of 2020 shows that the number of patients with CLDs, including chronic hepatitis, fatty liver, and cirrhosis, may exceed 447 million in China. The liver is a crucial organ for the metabolism of various substances, including sex hormones and lipids. CLDs frequently result in abnormalities in the metabolism of sex hormones, glucose, and lipids, as well as mental and psychological illnesses, all of which are significant risk factors for erectile dysfunction (ED). It has been reported that the prevalence of ED in male patients with CLDs ranges from 24.6 to 85.0%. According to a survey of Caucasians, liver transplantation may improve the erectile function of CLDs patients with ED. This finding supports the link between CLDs and ED. In addition, ED is often a precursor to a variety of chronic diseases. Given this correlation and the significant prevalence of CLDs, it is important to evaluate the epidemiology, risk factors, etiology, and treatment outcomes of ED in male patients with CLDs, expecting to attract widespread attention.

## 1. Introduction

Erectile dysfunction (ED) in men is a common disease with a high incidence. Although more common in the middle-aged and elderly, ED can occur in mature men of any age, and its incidence increases with age (1). It was reported that about 23% of men aged 40–80 worldwide suffer from ED to varying degrees (2). In mainland China, the overall incidence of ED is ~ 49.69% (3). Additionally, the information currently available points to an increase in the prevalence of ED in men younger than 40 years of age (1). Therefore, ED is pervasive across all ethnic groups and is becoming a bigger concern globally (4). The etiology and pathogenesis of ED are extremely complicated. It is widely accepted that spiritual and psychological factors, as well as some clinical factors mostly related to neurological, vascular, hormonal levels, and some drugs, contribute to being the main causes of ED. Overall, diabetes mellitus, cardiovascular illness, and neurological disorders are the main risk factors for ED clinically.

Chronic liver diseases (CLDs) are also common in clinical practice. As of 2019, an estimated 1.69 billion people worldwide were affected by liver disease (5). According to 2020 data, the number of patients with CLDs, including cirrhosis, fatty liver, and chronic hepatitis, may exceed 447 million in China. The liver is crucial for the metabolism of many substances, including sex hormones and lipids. Disorders of sex hormone metabolism, glucose and lipid metabolism, as well as mental and psychological conditions, are all common adverse complications of CLDs and high-risk factors for ED. According to studies, the prevalence of ED in male patients with CLDs ranges from 24.6 to 85.0%, including 8.6–78.0% in patients with chronic hepatitis and 41.2–92.0% in patients with cirrhosis. These estimates may greatly understate the true occurrence because several CLD patients are focused on treating their “dangerous” liver illness and lack the time and energy to concentrate on this “slight” issue. Several surveys indicated that the erectile function of CLDs patients with ED might improve after liver transplantation. Significant differences were found in total testosterone, sex hormone-binding globulin, free androgen index, and the International Index of Erectile Function-5 (IIEF-5) scores pre- and post-operation (6, 7), which confirmed an association between CLDs and ED. The simplified IIEF-5 score is the most commonly used tool to assess the presence and severity of ED, mainly including erectile and orgasmal function, libido, sexual intercourse, and overall satisfaction, and is also the most reliable and objective tool (8). Based on the IIEF-5 score, the severity of ED is divided into three categories: a total score of 22–25 points is normal, 12–21 is mild, 8–11 is moderate, and 5–7 is severe. For the assessment of liver function, currently, the modified Child-Pugh score is mostly used to quantify the liver reserve function of patients, which categorizes liver function into three levels based on the total score: a total score of 5–6 points is clarified as degree A, 7–9 as degree B, 10 points or more as grade C.

Currently, the majority of attention to the risk factors of ED is concentrated on diabetes mellitus, cardiovascular disease, neurological disease, and psychological factors. Few studies have evaluated the prevalence and risk factors for ED in patients with CLDs, and the attention given by physicians to this symptom in the diagnosis and treatment of liver disease is still poor. Given this correlation and the significant prevalence of CLDs, it is important to evaluate the epidemiology, risk factors, etiology, and treatment outcomes of ED in male CLDs patients in order to garner public interest. Those underlying causes of ED are not the main content of this review, we will integrate the up-to-date evidence and concentrate on the correlations, pathogenesis, and treatment status between CLDs and ED. This paper will cite a substantial amount of evidence to illustrate this issue, and these evidences, which are gathered from various literatures, varies to some extent and is frequently not comparable, possibly as a result of the various etiologies of liver disease, the sample size, the survey and patient sampling methodologies, and the assessment tools employed in each study.

## 2. Epidemiology of ED in CLDs

CLDs is a general term for a group of diseases characterized by progressive necrosis of hepatocytes, which leads to liver dysfunction. Recent research has confirmed that a variety of liver diseases can result in ED, particularly for those suffering from advanced liver disease. Multifactorial diseases are highly related to the existence of ED, which is not just a comorbidity, but liver disease *per-se* also leads to the progression of ED.

### 2.1. Non-alcoholic fatty liver diseases

NAFLD refers to a group of diseases primarily characterized by macrovesicular steatosis of the liver that occurs in patients without excessive alcohol consumption (9). Currently, it is considered to be the most common CLDs worldwide, especially in the Western, with an incidence of nearly 25% (10). The actual prevalence of NAFLD may be higher because many remain asymptomatic in the early stages and there are no reliable non-invasive tests for screening. Its primary feature is fat deposition in hepatocytes, which can develop into fibrosis, cirrhosis, and even hepatocellular carcinoma. The incidence and mortality of hepatocellular carcinoma and cirrhosis brought on by NAFLD have kept increasing in recent years (11).

NAFLD has been regarded as the hepatic manifestation of systemic metabolic syndrome (12, 13). ED is a very important and common comorbidity of NAFLD, some patients even have abnormal semen parameters. The incidence of ED in patients with NAFLD ranges from 45 to 67% (14), which is much higher than the rate in the general population and 2.92 times higher than the rate in those without NAFLD. The degree of fat infiltration is closely related to ED. NAFLD is a significant independent factor associated with ED. The main reason may be that NAFLD and ED share common risk factors such as obesity, hypertension, and diabetes mellitus (15, 16). Numerous ED etiologies are present in 39.5% of NAFLD patients, and a significant portion of these patients have psychogenic ED. Therefore, careful evaluation of psychological status during diagnosis and treatment is important (14).

### 2.2. Cirrhosis

Cirrhosis is a chronic and progressive liver disease commonly seen in clinical, which is caused by long-term or repetitive impacts of one or more etiologies. It is often accompanied by ascites, hepatic encephalopathy, and varicose hemorrhage.

A high prevalence of ED is shown in alcoholic hepatitis, which is one of the major etiologies of cirrhosis. At present, some scholars consider that there is a correlation between cirrhosis and ED, which can be explained by haemodynamic alteration, hypogonadism, hypotestosteronemia, unhealthy lifestyle, and a lower quality of life. Through meta-analysis, Yoo et al. (17) found that the prevalence of ED in cirrhotic patients was 79.08%, and in decompensated cirrhotic patients was 88.4%. After controlling for other conditions such as diabetes, alcoholism, severe cardiac conditions, etc., Paternostro et al. (18) discovered that among male cirrhotic patients, 55% were categorized as mild-to-moderate ED and 8.3% as moderate-to-severe ED, and that with the decline of liver function, the severity of ED increased and the IIEF-5 score decreased significantly. In cirrhotic patients, the increase in absolute level of hepatic venous pressure gradient is an independent predictor of ED, suggesting that ED is correlated with portal hypertension, and this may be related to hemodynamic alternation in the splanchnic circulation, which can directly impair physiological penile erection. In men with alcoholic cirrhosis, ED may be related to the direct toxic effects of ethanol and acetaldehyde on the gonads.

However, previous studies had shown conflicting results regarding the impact of cirrhosis on the prevalence and severity of ED. Some scholars compared the incidence of ED among alcoholics with or without cirrhosis and diabetes, found that there was no difference between the groups (19). According to some studies, no difference was found in the prevalence of ED between patients with chronic viral hepatitis and patients with established cirrhosis (20), only age and hypoalbuminemia were found to be independent factors for ED (18). The reasons for these disparities could be that these studies used different IIEF-5 cut-offs or that these patients had higher expectations of erectile function.

### 2.3. Chronic viral hepatitis

Chronic viral hepatitis is a common disease worldwide. Despite the promotion of vaccination, the landscape is still far from satisfactory, with more than 300 million people suffering from various kinds of hepatitis. According to the World Health Organization, an estimated 257 million people are living with chronic hepatitis B, more than 70 million with hepatitis C, 80% of whom develop chronic disease, and 62 million with chronic hepatitis D. As for hepatitis A, 1.4 million new cases are reported globally each year (21–24).

The total prevalence of ED in patients with chronic viral hepatitis ranges from 14 to 78% (20, 25), even after adjustment based on IIEF-5 scores ≤ 17, 40% of patients with chronic viral hepatitis had ED (26). According to the IIEF cut-off, ED was confirmed in 76.4% of hepatitis B patients, with 60.3% classified as severe. Those with diabetes mellitus had the highest incidence of ED (92.6%), followed by those who progressed to cirrhosis (85.7%) (27). Patients with HBV-related liver cirrhosis exhibited a higher prevalence and severity of ED than chronic hepatitis B patients (4, 28). In comparison to patients with HBV infection, those with HCV seem to be more susceptible to ED. This may be due to the distinct biological properties of these two viruses. HCV infection is more likely to cause inflammation of the vascular endothelium, which is one of the pathogenesis of ED (29, 30). Cryoglobulinemia may play an important role in this process. HCV infection is one of the causes of cryoglobulinemia; cryoglobulins depositing in the vascular endothelium can result in systemic vasculitis, primarily involving the small and medium arteries. HCV patients with cryoglobulinemia have a higher incidence of ED than those without cryoglobulinemia (31).

### 2.4. Alcoholic liver diseases

Alcoholic liver diseases are caused by long-term alcohol abuse and usually manifest as fatty liver at the initial stage and may develop into alcoholic hepatitis, liver fibrosis, and cirrhosis. Long-term alcohol abuse is generally defined as a history of drinking alcohol for more than 5 years, which is equivalent to ethanol ≥ 40 g/d for men and 20 g/d ≥ for women, or a history of heavy alcohol consumption within 2 weeks, which is equivalent to ethanol > 80 g/d. There have been no specific reports retrieved on the association between alcoholic liver disorders and ED, and the points of view we discussed ut infra are all obtained from sporadic observations in other studies, and the available data are inadequately standardized and the cases are restricted.

Cornely et al. (19) observed that the prevalence and severity of ED were considerably higher in male cirrhotic patients with chronic alcohol abuse than in patients with more severe cirrhosis due to other etiologies. Another study found that the prevalence of sexual dysfunction (particularly ED and/or decreased libido) in alcohol-induced liver disease is comparable between cirrhotic and non-cirrhotic patients, leading to the conclusion that alcohol was the true cause of ED (32). However, the results from another study were completely different, Wang et al. (33) believed that the history of alcohol consumption had no effect on the prevalence of ED in patients with cirrhosis, but the levels of testosterone, estradiol, and PRL were different from those in the control group, suggesting that cirrhosis rather than alcohol was the real cause of sexual dysfunction.

## 3. Etiology

### 3.1. Overall health status

#### 3.1.1. Age

For patients with CLDs, age often predicts a longer duration and more serious condition. In their study of 120 patients with Child-Pugh A cirrhosis, Maimone et al. discovered that the presence of ED was significantly associated with age, whether using univariate logistic regression analysis or multivariate analysis, and age was identified as an independent predictor of ED. The mean age of ED patients was significantly higher than that of non-ED patients, and the prevalence of ED gradually increased with age. Additionally, the severity of ED was positively correlated with age (34). In a Japanese study, 64 patients with chronic hepatitis and 53 patients with cirrhosis were studied with IIEF-5, and it was discovered that ~50% of patients under the age of 50 developed ED, ~79% of patients aged 50–59 years old had ED, and all patients over the age of 60 had ED (4).

#### 3.1.2. Diabetes mellitus

Diabetes mellitus is a common comorbidity of CLDs. Persistent hyperglycemia can affect erectile function in multiple ways. First, it can negatively affect endothelial function, resulting in an imbalance between NO and endothelin-1, which causes relaxation disorder of smooth muscle cells (35); second, it can increase the levels of advanced glycosylation end products, which can interfere with the synthesis of protein polymerase chains and may directly affect DNA replication and transcription, ultimately leading to the atherosclerosis and stenosis of small and medium arteries, resulting in penile hypoperfusion and ED (36); third, the accumulation of advanced glycosylation end products can directly damage the structure and function of corpus cavernosum smooth muscle cells; fourth, a hyperglycemic state can damage the peripheral and autonomic nerves *via* the polyolol pathway, the protein kinase C pathway, and by competitively inhibiting inositol extraction by neural tissue (37, 38). Furthermore, insulin resistance can impair gonadal axis function. All of these factors could contribute to diabetes-related ED.

#### 3.1.3. Hypertension

It is well established that hypertension can elevate the risk of ED. This could be due to hypertension itself or antihypertensive medications. In patients with chronic hepatitis B and cirrhosis, hypertension is an important independent factor for ED (18), and ED can also be used as an early marker of endothelial dysfunction in hypertension (39). Endothelial dysfunction and nitric oxide (NO) play important roles in the occurrence and development of ED in patients with hypertension (40, 41). Moreover, some antihypertensive medications, such as beta-blockers, aldosterone receptor antagonists, and thiazide diuretics, can also induce ED (42).

#### 3.1.4. Mental state

Depression and anxiety are associated with impaired sexual function and satisfaction and have a high prevalence in patients with chronic viral hepatitis (43–45), and they have an independent negative effect on erectile function in these patients (26). The incidence of concomitant depression was also higher in patients with cirrhosis than in the general population (46). Depression appeared to have a stronger correlation with sexual dysfunction than testosterone levels (47). Mental disorders can contribute to the development of ED by reducing libido and physical activity (26). In addition, the adverse side effects, such as psychosocial problems caused by depression and antidepressants, may aggravate ED. Although the precise mechanism has not been clarified, it is proposed that depression and anxiety contribute to a vicious cycle that impairs the sexual relationship between patients and their partners, resulting in communication problems that further impede sexual functioning (48). Therefore, in patients with CLDs, active detection and intervention should be carried out to reduce the occurrence or severity of ED.

### 3.2. Etiology and severity of liver disease

The etiology and progression of liver disease contribute to the development of ED. Toda et al. (4) studied 117 subjects with viral hepatitis and idiopathic non-alcoholic liver disease, 53 of whom had cirrhosis (30 Child A, 17 Child B, and 6 Child C), and assessed their erectile function with the IIEF-5, finding that the incidence of ED increased as the Child-Pugh score worsened. However, in patients with Child-Turcotte-Pugh scores ≤ 10, their frequency and severity did not vary with the severity of liver disease.

Hepatitis viruses can directly damage the gonads and can also affect the occurrence of ED through a variety of complex mechanisms such as inflammation, oxidative stress, and apoptosis (25, 26). Chronic systemic inflammation with elevated C-reactive protein levels reduces the synthesis of NO in endothelial cells and ultimately leads to endothelial dysfunction, which may account for the relationship between ED and chronic hepatitis (49, 50). Compared with HBV, HCV infection had a more pronounced negative effect on erectile function; compared to the control group without infection, men with chronic HCV infection had significantly lower libido, erectile function, ejaculation, and overall satisfaction (51). Even after controlling for depression or other potentially confounding variables, the association between HCV and ED remains strong. This could be due to biological/virological factors or potential effects on the hypothalamic-pituitary-gonadal axis (52). The prevalence of ED in cirrhotic patients is higher than in chronic hepatitis patients. The mechanism remains unclear, but gonadal dysfunction, sex hormone imbalance, and low albumin levels may be the major causes (27). Kim M et al. (28) also found that the incidence of ED was significantly higher in patients with HBV-related cirrhosis than in chronic hepatitis patients without cirrhosis, and this difference remained significant even after four patients with Child-Pugh grade B cirrhosis were excluded. Physiological disturbances caused by protein malnutrition in patients with decompensated CLDs may be associated with ED. Edema, ascites, hypoalbuminemia, pleural effusion, and deteriorating physical function are usually present in advanced liver diseases and can also reduce libido and lead to ED. Branched-chain amino acids can improve serum albumin, muscle metabolism, and prognosis in patients with CLDs (53). In cirrhotic patients treated with branched-chain amino acids, an improvement in erectile function was observed (54), suggesting a correlation between physiological dysfunction and ED. Portal hypertension, as an independent risk factor for erectile dysfunction, can impair penile erectile function by altering the hemodynamic status of the visceral circulation (18). In a study of cirrhotic patients with a wider range of liver failure, Phylonenco et al. identified minimal hepatic encephalopathy as a possible risk factor for the progression of ED (55).

Minimal hepatic encephalopathy is considered to be associated with a poor quality of life as well as some behavioral abnormalities such as depression and anxiety. In their study, Nardelli et al. (56) found that the prevalence of ED was significantly higher in patients with abnormal neurocognitive tests than in normal subjects, and that, in the subgroup of patients with Child-Pugh A cirrhosis (*n* = 25), 100% of patients with minimal hepatic encephalopathy (*n* = 16) developed ED. However, in multivariate analysis, it was not identified as an independent risk factor for ED. This could be due to the sample size restrictions, which make it challenging to distinguish the effects of the two variables, liver dysfunction and cognitive impairment (57). Huyghe et al. (58) used a questionnaire to survey patients with end-stage liver disease who were candidates for liver transplantation, using the IIEF-5 score to assess erectile function and the patient-baseline Treatment-Satisfaction Scale score to assess sexual satisfaction. Of the 98 candidates who completed the questionnaire, 28 (29%) were sexually inactive, while 52 (74%) of the 70 sexually active patients had ED. Approximately 50% of patients felt that their erectile function had deteriorated in the previous 6 months.

In addition, some rare diseases can also cause liver dysfunction and ED. Hemochromatosis is a chronic iron overload caused by a high-iron diet, massive blood transfusions, or systemic disease. Excess iron stored in substantial cells such as the liver, heart, and pancreas can lead to degeneration and diffuse fibrosis, as well as metabolic and functional abnormalities. It can also cause hypothalamic-pituitary-gonadal axis dysfunction, direct testicular damage, diabetes, and other conditions that can lead to sexual dysfunction (59, 60). Autoimmune hepatitis is a chronic and progressive autoimmune-mediated inflammatory disease of the liver. Approximately 34% of patients have no symptoms but abnormal liver function at the initial visit. Thirty percent have cirrhosis, and 8% have decompensated cirrhosis symptoms such as hematemesis and/or melena. It may be connected to other autoimmune conditions like diabetes, which can result in hormonal imbalance and disruption of the hypothalamic-pituitary-gonad axis, ultimately impairing sexual function (61).

## 4. Mechanisms of CLDs related ED

### 4.1. Gonadal dysfunction

The liver is the largest metabolic organ in the human body, and it is also the primary site of sex hormone metabolism. It is well known that CLDs patients exhibit significant feminization, including gynecomastia, fat redistribution, loss of body hair, and other symptoms, as a result of hypogonadism and excessive estrogen production (8). Up to 90% of patients with cirrhosis have lower testosterone levels (62), which correlate with disease severity, and these patients tend to lose their circadian rhythm in testosterone levels. In the compensatory stage of liver disease, sex hormones may not change noticeably, but as liver function deteriorates to a decompensated stage, patients may present with testicular atrophy and interstitial fibrosis, which reduce the synthesis and secretion of testosterone.

The precise mechanisms underlying the associations between gonadal dysfunction and CLDs remain poorly understood. Testicular atrophy is present in more than 50% of male patients with cirrhosis. It may manifest histological abnormalities such as atrophy of the testis germ epithelium, thickening of the tubule basement membrane, and fibrosis of the leydig. In addition to testicular atrophy, CLDs may cause gonadal dysfunction *via* a variety of mechanisms. As is well known, as liver disease progresses, the liver's inactivation effect on estrogen weakens, and the transformation of testosterone and estrone into estradiol increases, leading to an increase in serum estradiol level, which inhibits pituitary gonadotropin release and reduces androgen secretion by testicular interstitial cells (62). In patients with NAFLD, the hypotestosteronemia can be explained by the hypogonadal-obesity-adipocytokine hypothesis (63). An increase in visceral adipose tissue raises the activity of the aromatase enzyme, which can convert testosterone to estrogen and decrease testosterone levels. Low testosterone levels then enhance the activity of the lipoprotein lipase enzyme, which promotes triglyceride uptake into adipocytes and further increases visceral adiposity, resulting in a vicious cycle (64). In addition, pro-inflammatory adipocytokines released from adipose tissue, including tumor necrosis factor-α, interleukin-1, and interleukin-6, have been shown to inhibit the pituitary axis, which in turn reduces testosterone levels (65). It had previously been demonstrated that ED caused by non-alcoholic steatohepatitis-induced testosterone deficiency was associated with increased TNF-α (66). The decreased activity of 17-β hydrogenase in the liver of CLDs patients reduces the conversion of androstenedione to testosterone, which is also one of the reasons for testosterone deficiency. However, what calls for special attention is that physiological decline should also be fully taken into account when analyzing testosterone levels in patients with CLDs, testosterone in men decays at a rate of 10% every decade after the age of 30 (67). ED may occur due to hormonal imbalances. Reduced testosterone levels can affect erectile function *via* multiple pathways, including increased apoptosis of smooth muscle cells and endothelial cells, resulting in decreased compliance and hemodynamic abnormalities of the corpus cavernosa; inhibition of the eNOS-No-cGMP pathway; and activation of the Rho A/Rho kinase pathway, resulting in decreased smooth muscle contraction and corpus cavernosum blood perfusion. In patients with cirrhosis, however, oral androgens may not increase serum testosterone levels but instead increase estrogen levels (8). Oral testosterone seemed to have no positive effect on sexual dysfunction in patients with cirrhosis, and androgen supplementation was only effective in cases of severe hypogonadism with testicular atrophy (51), implying that the main mechanism for ED may not be limited to total testosterone levels.

Although total testosterone levels are significantly lower in CLDs patients with ED, they were not significantly associated with the occurrence and severity of ED in multivariate analyses, raising the question of whether total or bioavailable testosterone is the more meaningful test method. Testosterone is mainly bound to albumin (50%), sex hormone-binding globulin (45%), and free testosterone (2%). Bioavailable testosterone includes albumin-bound testosterone and free testosterone. According to a meta-analysis, free testosterone levels were significantly lower in ED patients than in the non-ED group (17). Patients with hepatic insufficiency frequently have higher levels of estrogen and lower levels of testosterone, which can result in an increase in the production of sex hormone-binding globulin through a negative feedback loop. That then results in a higher binding between testosterone and sex hormone-binding globulin, further reducing bioactive free testosterone (62, 68). A hypothesis was proposed that low serum albumin could affect the ratio of free albumin to bound testosterone, thereby altering the testosterone response (51). In conclusion, free or bioactive testosterone may be a better marker of ED.

### 4.2. Drugs or medications

Drugs or medications can induce or aggravate ED in patients with CLDs.

#### 4.2.1. Alcohol

Alcohol abuse is a well-known risk factor for the development of alcoholic cirrhosis (69), as well as a risk factor for ED in the general population (70). In alcoholic and non-alcoholic cirrhosis, the prevalence of ED is 70 and 25%, respectively. Alcohol can cause nutritional metabolism disorders and damage to multiple systems and organs, such as the gonads and nerves, altering the balance between linked and available steroid hormones, with the final effect being detrimental to normal sexual function. Martinez-Riera et al. (71) found that the levels of basal SHBG, androstenedione, estradiol, and prolactin in patients with alcoholic liver disease were higher than those in the control group, regardless of the presence of cirrhosis, and tended to increase the levels of FSH and LH. Abstaining from alcohol is effective for the recovery of gonadal dysfunction to a certain degree if these patients do not have testicular atrophy or a poor response of gonadotrophins to luteinizing hormone-releasing factor or clomiphene. A decreased responsiveness to androgens in alcohol addicts has not been verified because the androgen receptors may present some structural alterations limiting regular function. But Maimone et al. (34) observed no significant relationship between the prevalence or severity of ED and alcohol consumption. This study, however, cannot rule out the association between alcoholic cirrhosis and ED. This could be due to the relatively small sample size of patients with alcohol-related cirrhosis and heavy drinkers in this study, or to multiple coexisting factors that offset the potential difference in the prevalence of ED based on alcohol consumption.

#### 4.2.2. Non-selective β receptor blockers

Non-selective β receptor blockers, such as propranolol and carvedilol, which are widely used to treat portal hypertension, have been shown to have a negative effect on erectile function (72), increasing ED by 2.32 times (18). The mechanism may be that non-selective β receptor blockers reduce adrenergic efflux by inhibiting angiotensin II, renin, and vasodilation (39).

#### 4.2.3. Antidepressants

Some patients with CLDs are accompanied by varying degrees of depression, which *per-se* can lead to ED. Chronic use of antidepressants, no matter monoamine oxidase inhibitors, tricyclic drugs, or selective serotonin reuptake inhibitors (SSRIs), can cause further exacerbation of ED symptoms. The incidence of ED varies with different SSRIs. A multicenter survey conducted by the United States of 1,763 male depressed patients treated with SSRIs found that the incidence of sexual dysfunction ranged from 7 to 30%, with the highest incidence of paroxetine (34%) and the lowest incidence of fluoxetine (7%) (73). This adverse side effect could be caused by serotonin, dopamine, acetylcholine, prolactin, NO, or another transmitter (74).

#### 4.2.4. Diuretics

Among diuretics, spironolactone has anti-androgenic properties due to its similar structure to sex hormones. Therefore, it can competitively inhibit dihydrotestosterone's binding to androgen receptors and enhance testosterone clearance, which in turn leads to decreased libido and ED.

#### 4.2.5. Interferon

Interferon, a commonly used medicine for the treatment of viral hepatitis, can cause patients to be depressed and prone to decreased libido and sexual dysfunction (75, 76). This malfunction generally returns to normal following the course of treatment and is not a negative side effect of interferon *per-se* (77). But there are too few studies to clarify the issue or figure out whether the condition is related to interferon-induced depression or specific hormonal changes.

In addition, immunosuppressive therapy with steroid agents such as azathioprine, cyclosporine A, or tacrolimus can affect the hypothalamic-pituitary-gonadal axis by inhibiting androgen synthesis, leading to ED in liver transplant patients (78). In the treatment of CLDs, the incidence of ED is higher when the drugs aforementioned are used in combination.

### 4.3. Insulin resistance

**Figure 1:**
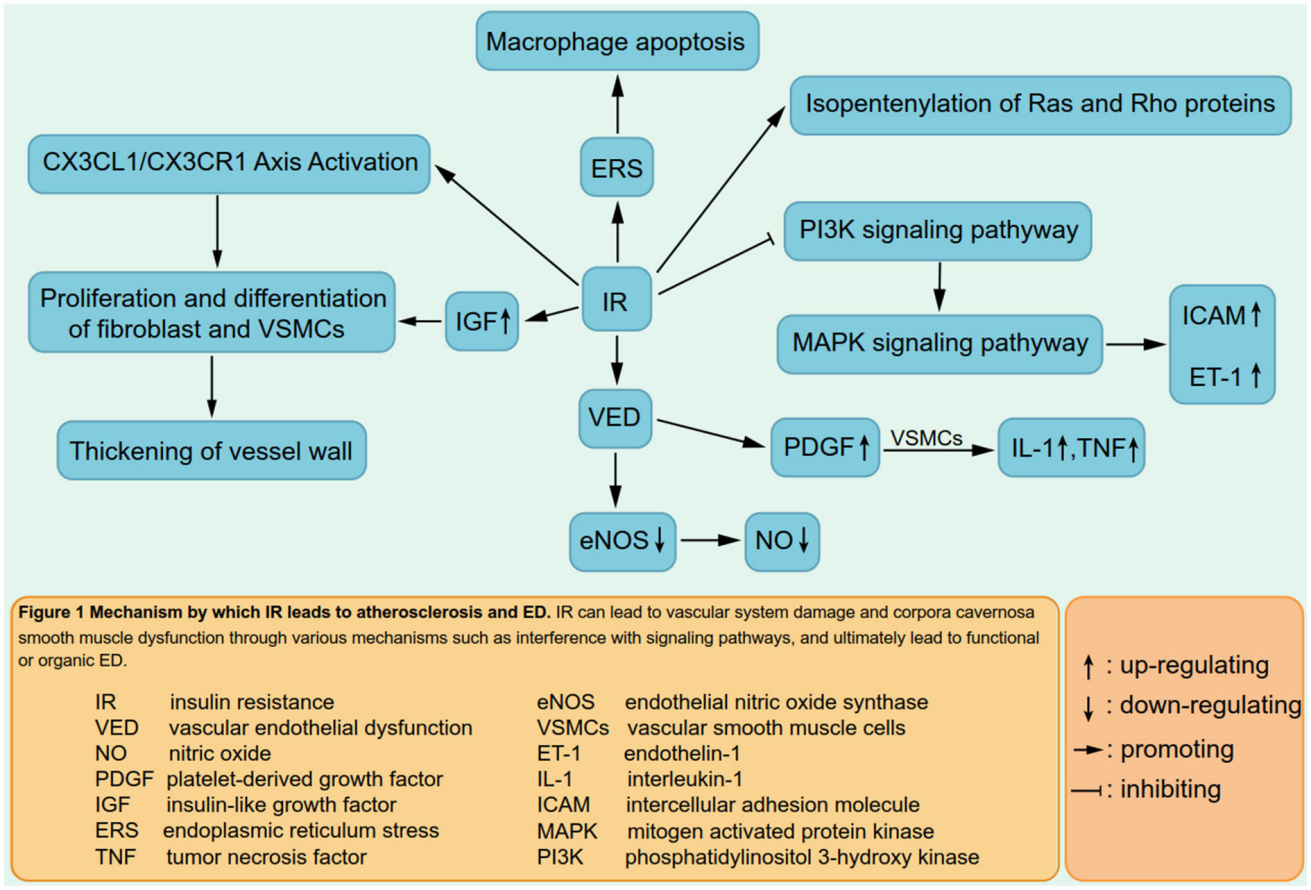
Mechanism by which IR leads to atherosclerosis and ED. IR can lead to vascular system demage and corpora cavernosa smooth muscle dysfunction through various mechanisms such as interference with signaling pathways, and ultimately lead to functional or organic ED.

IR is associated with hepatic fat deposition and is significantly increased in patients with NAFLD (79). The accumulation of lipid oxidation products in cells may interfere with the insulin signaling pathway *via* the phosphatidylinositol 32 kinase (PI32K), thereby affecting the transport function of glucose transporter 4, resulting in IR. IR can harm the vascular system through a variety of mechanisms, causing vascular dysfunction or structural destruction, such as atherosclerosis (80, 81), and eventually leading to ED (Figure 1). In the meantime, eNOS deficiency aggravates the early stage and accelerates the progression of NAFLD (82). This is a vicious circle, and it could be one of the mechanisms by which NAFLD leads to ED.

### 4.4. Anemia

Anemia is common in patients with decompensated CLDs, and ED is also associated with lower hemoglobin levels. Low serum hemoglobin levels may indicate advanced liver disease and severe portal hypertension. Anemia can exacerbate the cirrhosis-related hyperdynamic circulation (34), which is characterized by visceral vasodilation and peripheral vasoconstriction. This might impair penile circulation and lead to ED (83). Furthermore, anemia reduces the supply of oxygen to tissues, including the corpus cavernosum, potentially impairing erectile function (84).

In addition to the aforementioned mechanisms, oxidative stress, inflammatory response, apoptosis, and other factors are also involved in the occurrence of CLDs-related ED (85, 86). Therefore, the mechanism of ED caused by CLDs is very complex and differs from that of the general population in some aspects. These distinct causes must be fully considered. Unfortunately, there are few studies on its concrete mechanisms, such as signaling pathways, that have been retrieved so far. More in-depth studies are needed to uncover the distinct mechanisms by which CLDs lead to ED.

## 5. Pharmacotherapy

There are few reports on the treatment of CLDs-related ED, the improvement of ED symptoms in these patients is mostly found during treatment of the primary disease, such as liver transplantation, and so on. Evidence on the efficacy and safety of using specific drug therapies to treat ED in this population is scant at best (87). The few existing studies have focused on phosphodiesterase type 5 (PDE-5) inhibitors, which are recommended as first-line medicines for the treatment of ED in men. We assume that the following are the primary causes of this situation: (a) Patients with CLDs are eager to solve their liver problems first and rarely consider or feel embarrassed to mention their erectile dysfunction problems while visiting hepatologists; (b) both patients and hepatologists are concerned about the adverse side-effects of related drugs on liver disease, as well as the contraindications of their compatibility with drugs for liver disease.

PDE-5 inhibitors increase intracellular cGMP concentration by inhibiting the activity of PDE-5, thereby improving the relaxation function of corpus cavernosum smooth muscle cells, resulting in smooth muscle relaxation, increasing the blood flow of the intracavernous arteries, and finally causing the penile erection. Sildenafil has been used for the treatment of porto-pulmonary hypertension in liver transplant patients in order to ensure the success of transplantation (88). Several studies have demonstrated the safety and efficacy of sildenafil citrate in patients with liver cirrhosis and porto-pulmonary hypertension, reporting that it did not affect portal pressure or hepatic venous pressure gradient in patients with compensatory liver cirrhosis, although it resulted in lower arterial pressure. These studies had also shown that PDE-5 inhibitors could be safely used in Child's A and B cirrhosis, and they might be considered as a treatment option for CLDs patients (89, 90), but these studies had not documented the efficacy in patients with cirrhosis. Thakur et al. (87) conducted a prospective study in which 25 cirrhotic patients with Child-Pugh scores between 5 and 10 were enrolled. After administration of 10 mg of Tadalafil per day for 4 weeks, IIEF scores were significantly improved in all patients, and 44% of the patients had complete resolution of ED. Those with lower IIEF scores and more severe ED showed the most significant improvement. The area under the curve of tadalafil in subjects with mild and moderate hepatic impairment is similar to that in healthy subjects, and tadalafil is well tolerated with few significant side effects, so it can be safely used in these patients without dose adjustment, but there is no sufficient evidence of its effect in patients with severe hepatic impairment (27). This is mainly because, for safety reasons, these patients have generally been excluded from randomized clinical trials. A study of severe hepatic dysfunction has been retrieved, patients with ED in compensated CLD and advanced fibrosis were treated with tadalafil orally, 20 mg on alternate days. It was discovered that erectile function improved significantly in all patients after 12 weeks of treatment, with only 25% of patients still having ED at 6 months of follow-up (91). However, there were some problems with this study. First, the sample size was small and the dropout rate was high, only 23 of the 34 patients completed the 3-month follow-up and only 8 completed the 6-month follow-up; second, and most importantly, the study did not indicate how many patients with severe hepatic impairment completed the study. A decrease in liver stiffness measurement and fibrosis index based on 4 factor values were also observed in this study, possibly due to the antifibrotic effect of tadalafil (92).

PDE5-I can protect the liver from ischemia-reperfusion injury through a variety of signaling pathways, such as increasing intracellular cGMP levels, activating protein kinase C and protein kinase G (93), leading to the activation of cGMP-dependent protein kinase, which in turn induces vasodilation, anti-inflammatory and anti-proliferative effects, and reduces collagen synthesis (94, 95). Pretreatment with tadalafil can protect against thioacetamide-induced liver fibrosis in a dose-dependent manner and reduce biomarkers of inflammation and fibrosis (96, 97). Therefore, the use of PDE-5 inhibitors in patients with severe hepatic insufficiency is theoretically beneficial.

Through a comprehensive analysis of the limited literature, we believe that PDE-5 inhibitors can be safely used in patients with mild to moderate hepatic impairment, and due to the limited information on the clinical safety of this drug in patients with severe hepatic insufficiency, a lower starting dose of PDE-5 inhibitors should be considered. To date, it is unclear which PDE-5 inhibitor is more effective. Although two meta-analyses suggested that tadalafil and sildenafil were equally effective (96, 97) and significantly better than vardenafil, another meta-analysis failed to show this significance (98). On account of its advantages in half-life and compliance, in particular, the pharmacokinetics of sildenafil citrate are affected by kidney and liver injury (91, 99), tadalafil may be more acceptable to clinicians and patients.

In the available literature, the most detailed protocol for the use of PDE-5 inhibitors according to the extent of liver function impairment comes from Neong and his colleagues (8). In order to improve the quality of life in the long term, larger studies and randomized trials are needed to further explore the efficacy, safety, and dosage of PDE-5 inhibitors in patients with CLDs. We also hope that other methods for treating ED, such as psychotherapy, combined androgen therapy, vacuum suction, functional electrical stimulation therapy, etc., will be applied to the study of CLDs-related ED so as to find more ways for these patients to improve their sexual satisfaction. In addition, the treatment of ED in patients with CLDs is challenging because it is often multifactorial and may require multidisciplinary involvement.

## 6. Conclusion

CLDs patients with ED are an overlooked group by both patients and doctors alike. At present, only a handful of related reports can be retrieved worldwide. Although findings vary from study to study and even conflict, most researchers believe that ED is common in patients with CLDs, irrespective of etiology, and that there is a correlation between CLDs and ED. Therefore, the erectile function and sexual satisfaction of CLDs patients should arouse the attention of clinicians. We strongly recommend a routine screen for ED in men with CLDs.

The mechanism by which liver damage affects penile erectile function is not fully understood. Through the analysis of literature results, we believe that this is multifactorial. Male patients with CLDs often have decreased testosterone levels and increased estrogen levels, as well as other sex hormone metabolism disorders. The resulting changes in penile tissue structure and the NO-cGMP pathway may be one of the mechanisms of ED in these patients. Age, liver function classification, hypohemoglobinemia, hypertension, diabetes mellitus, alcohol consumption, mental state, and certain therapeutic medicines may be risk factors for ED in patients with CLDs. While treating the primary disease, we should actively remove the risk factors to reduce the occurrence of ED.

There are few reports on the treatment of ED associated with CLDs. In addition to the treatment of the primary disease, short-term, low-dose PDE-5 inhibitors are safe and reliable. Whether combined androgen therapy can increase its efficacy is still controversial. We look forward to more relevant studies to provide data for reference.

## Author contributions

The epidemiology, mechanisms, pathophysiology, and treatment of ED in CLDs patients are discussed in detail by all the authors. All the authors we list have made direct or indirect contributions to this work and approve its publication.
